# Supervisor Developmental Feedback and Voice: Relationship or Affect, Which Matters?

**DOI:** 10.3389/fpsyg.2019.01755

**Published:** 2019-08-13

**Authors:** Zhenduo Zhang, Li Zhang, Junwei Zheng, Bao Cheng, Vivi Gusrini Rahmadani

**Affiliations:** ^1^School of Management, Harbin Institute of Technology, Harbin, China; ^2^Department of Construction Management, Kunming University of Science and Technology, Kunming, China; ^3^School of Management, Xiamen University, Xiamen, China; ^4^Faculty of Psychology and Educational Sciences, KU Leuven, Leuven, Belgium

**Keywords:** supervisor developmental feedback, voice, positive affect, perceived rapport, social exchange theory

## Abstract

Employee voice is the discretionary communication of ideas, suggestions, or concerns to benefit the organization. Employee voice is important for both organizations and employees. As such, this study examined the relationship between supervisor behavior and voice, by exploring the positive influences of supervisor developmental feedback on employee voice at the episode level. Further, this study explored the underlying mediators of positive affect and perceived rapport in the relationship between supervisor developmental feedback and employee voice, based on social exchange theory. The study collected 310 matched data points, collected across 62 employees for five consecutive days, using an experience sampling method with mobile surveys. Day-level supervisor developmental feedback positively related to day-level employee voice. Positive affect and perceived rapport with supervisors mediated the relationship between supervisor developmental feedback and employee voice at the episode level. The findings extend the antecedents of voice and examined the social exchange process at a within-person level.

## Introduction

Employee voice is the discretionary communication of ideas, suggestions, or concerns intended to benefit the organization (Morrison, 2011). It is believed to play a critical role in enhancing organizational effectiveness and promoting employee development (Mowbray et al., 2015). Considering the importance of voice for both organizations and employees, most voice research has focused on exploring the antecedents of employee voice. Morrison (2014) suggested that a latent voice opportunity is the starting point for employee voice. Whether or not an employee renders his or her voice depends on the inherent desire or motivation to bring about a constructive change for the organization.

Within a social exchange theory (SET) framework, past research has examined the positive relationship between leadership and employee internal voice motivation (Detert and Burris, 2007). The expectation that employees will exercise their voices depends on supervisors showing support (Lin et al., 2017; Ye et al., 2019). When supervisors convey concerns for employee improvement, employees develop the belief that their organization has a positive orientation toward their growth in organizations. This, in turn, increases the probability that employees will participate in social exchanges and share their beneficial ideas to facilitate organization effectiveness (Tucker et al., 2008; Mo and Shi, 2018). This is because a supervisor is an organization’s representative (Eisenberger et al., 2002).

Previous research examined voice trajectories over longer time periods (Li and Sun, 2015; Zhang et al., 2015). However, short-term tends have not been addressed (Morrison, 2011). Liu et al. (2017) and Starzyk et al. (2018) provided evidence for the possible fluctuations at the episode level. This highlights the importance of exploring the antecedents of voice by adopting a within-person approach and provides a more dynamic picture of employee voice (Morrison, 2011). Within a daily organization context, supervisors play critical roles as information providers to their employees (Abu Bakar et al., 2010). Feedback is a basic strategy of a leader interacting with employees (Mayfield and Mayfield, 2007). Zhou (2003) proposed the concept of supervisor developmental feedback, which refers to the extent to which supervisors provide helpful or valuable information to their employees. This feedback supports on-the-job learning, development, and improvements. Previous research examined the influence of supervisor-provided feedback on enhancing employee creativity and in-role performance (Zhou, 2003; Li et al., 2011).

This study examined supervisor developmental feedback as a critical antecedent to employee voice, because it provides a resolution for employee concerns that they must “read the wind” to discern whether it is appropriate to express voice with their leaders in a specific situation (Milliken et al., 2003). Supervisor developmental feedback offers employees a clear guide for how to behave in organizations. This feedback helps create a positive atmosphere free from pressure, facilitating employee willingness to express voice (Li et al., 2011).

The present study draws on SET (Cropanzano and Mitchell, 2005). SET contends that leader behavior is a social influence process, through which emergent reciprocal cooperation and consequential beneficial changes are socially developed (Uhl-Bien, 2011; Chun et al., 2016). SET is relational in nature (Cropanzano and Mitchell, 2005). Workplace relationships is the aspect of SET that has garnered the most research attention (Cropanzano and Mitchell, 2005). Usually, relationship is considered the main reason why employees render positive work behavior, returning supportive gestures from supervisors Lawler and Thye (1999), Lawler (2001) introduced affect into SET. He argued that employees’ daily feelings are intertwined with social exchange. Emotions are subtle signals to employees about their responses based on their interactions with supervisors. Furthermore, Colquitt et al. (2013) regarded relationship and affect as two distinct paths in social exchange based on a meta-analysis. Based on this logic, we adopted two key dimensions to elaborate how daily supervisor developmental feedback influences employee voice. The first was perceived rapport, which is the employees’ subjective perceptions of outcomes of interaction with their supervisors, including the viewpoint that one has been justly heard and treated. This perspective relates to the positive impressions and trust that employees have toward their supervisors (Curhan et al., 2010). The second perspective is positive affect, which is the extent to which a person feels alert, active, and enthusiastic (Watson et al., 1988). We assume that the supervisor provides developmental feedback to the employee. This enhances employees’ perceived rapport and positive affect, strengthening their reciprocal motivation and ability to engage in voice.

**FIGURE 1:**
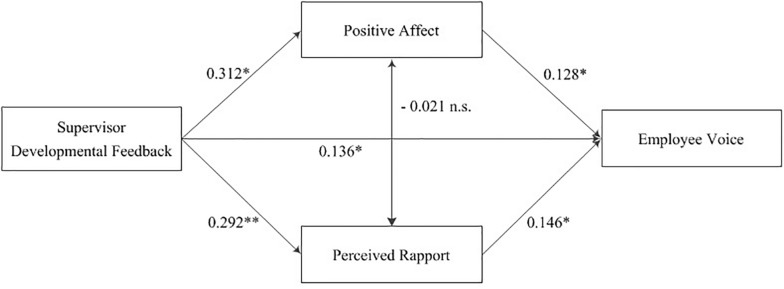
Conceptual model.

We conducted a multi-wave diary study to test our theoretical model of whether and how supervisor developmental feedback benefits employee voice (Figure 1). This research contributes to the voice and SET literature in two ways. First, by focusing on the positive influences of daily supervisor developmental feedback, we extend previous research on the relationship between supervisor behavior and employee voice. Previous research has provided fruitful results related to this relationship. However, it remains unclear how a supervisor interacting with employees affects voice. Feedback is a basic interaction strategy for supervisors, conveying important information relevant to employee development and career growth in organizations (Moss and Martinko, 1998). This research explores the episodic influence of supervisor developmental feedback on employee voice, extending our understanding of the antecedents of employee voice.

Second, by uncovering the dynamic mediating role of perceived rapport and positive affect, we contribute to the development of SET. Previous social exchange research has been conducted mainly at the between-person level (Aryee et al., 2015; Yan et al., 2016). However, social exchange is a dynamic process, and employee perceptions about their supervisors are not stable (Ellis et al., 2018). Gaining insights into the possible episodic fluctuations in a beneficial social exchange process has important implications for the long-term maintenance of the positive influences of supervisor behavior and the extension of sustainable employee proactive work behavior over time (Neff and Karney, 2009). Our research highlights the underlying mechanism through which supervisor developmental feedback affects employee voice, by unveiling the mediating role of perceived rapport and positive affect at the episode level. Using this approach, this research offers a dynamic social exchange process in organizations.

## Hypothesis Development

### Supervisor Developmental Feedback and Employee Voice

The definition of supervisor developmental feedback provided by Zhou (2003) suggests that it includes three specific characteristics. First, feedback is informational in nature. Supervisor developmental feedback is useful and provides valuable information for employees, facilitating improvements on the job. Second, supervisor developmental feedback is future-oriented, focusing on the employees’ growth in the organization, rather than on the discrepancies between anticipated goals and achieved outcomes. Third, developmental feedback is well suited to creating a positive team climate, free from fear of making mistakes (Zhou, 2003; Li et al., 2011).

Employee voice is risky and costly, because it challenges the *status quo* and places stress on existing workplace relationship (Ng and Feldman, 2012). Previous research has indicated that whether employees render voice or not depends on supervisors’ supportive behavior (Zhang et al., 2015). Supervisor developmental feedback reveals supervisors’ genuine care and attention, which nurtures employees’ sense of gratitude and indebtedness. This ensures a reciprocal interpersonal relationship and further motivating them to voice to provide change-oriented suggestions to the organizations as a way of reciprocating for the positive treatment they have received from their supervisors (Carnevale et al., 2017). Furthermore, the agreeable team climate created by supervisor developmental feedback strengthens employee psychological safety, which has been examined as a critical motivator of employee voice. Research has provided firm empirical evidence for the relationship between supervisor support and employee voice within social exchange framework (Liang et al., 2012; Liu et al., 2017). For example, Carnevale et al. (2017) argue that a supervisor facilitates employees’ proactive attempts to express their voice by offering valuable resources. Thus, we hypothesized the following:

Hypothesis 1. Supervisor developmental feedback (morning) relates to day-level employee voice.

### Mediating Role of Perceived Rapport With Supervisors

#### Supervisor Developmental Feedback and Perceived Rapport With Supervisors

Perceived rapport is the immediate subjective outcome of interaction with the supervisor (Curhan et al., 2006). Rapport is composed of two factors: feelings about the process and feelings about the relationship. Feelings about the process include perceptions that the employees have been justly treated and heard, and that the interaction process with supervisors is efficient. Feelings about the relationship include the positive impressions of and trust toward the supervisors. This creates a solid foundation for working and interacting with the supervisors in the future (Curhan et al., 2010).

Social exchange theory is relational in nature (Cropanzano and Mitchell, 2005). Previous research has adopted leader–member exchange (LMX) to indicate the relationship between supervisor and employee in the social exchange process (Reb et al., 2018). LMX is a two-way interaction, in which a supervisor and an employee deliberately exchange tangible or intangible resources relevant to work tasks and social intentions (Sheer, 2015). Given the daily supervisor–subordinate interaction context, subordinates form perceptions of relationship quality with supervisors based on the subjective experiences. This is the dynamic foundation for LMX (Selvarajan et al., 2018). This study’s goal was to examine the underlying mechanism through which supervisor developmental feedback influences employee voice at the episode level. As such, the study adopted perceived rapport, instead of LMX, as the proxy mediator between supervisor developmental feedback and employee voice.

Based on the arguments above, we proposed that supervisor developmental feedback influences perceived rapport by enhancing feelings about the process and feelings about the relationship. Feelings about the process involve employees’ perceptions of procedural and interpersonal justice with respect to communication with supervisors (Curhan et al., 2006). SET suggests that employees tend to recognize the quality of treatment received through exchanges with supervisors as an indicator of perceived justice (Lee and Jensen, 2014). The provision of feedback to employees is an important process, as it allows supervisors to influence employees’ perceptions of justice (Karkoulian et al., 2016).

Supervisor developmental feedback is future-oriented, focusing on employee improvement on the job (Zhou, 2003). Developmental feedback reveals a supervisor’s care for an employee’s career growth. It involves devoting valuable resources to reward appropriate behavior (Zhou, 2003). As such, employees will feel respect and that they are treated with sensitivity when receiving supervisor developmental feedback. This strengthens their perceptions of justice toward the communication process.

Feelings about the relationship include positive impressions of and trust toward supervisors. Employees develop trust toward their supervisors when they attribute the supervisors’ behavior to sincere motives and selflessness (Chen et al., 2014). Further, they develop positive impressions toward their supervisors, based on the supervisors’ demonstration of character. Developmental feedback reveals supervisors’ expressions of sincere and holistic concern about their employees’ personal welfare. Thus, supervisor developmental feedback is likely to trigger employees’ gratitude and indebtedness, ensuring reciprocal supervisor–employee relations and firm affective bonds of trust. Thus, we hypothesized the following:

Hypothesis 2. Day-level supervisor developmental feedback (morning) positively relates to day-level perceived rapport.

#### Perceived Rapport With Supervisors and Employee Voice

Trust toward supervisors and perceptions of justice are two core factors associated with perceived rapport (Curhan et al., 2010). Previous research has shown a positive relationship between perceived rapport and sequential active behavior (Curhan et al., 2009).

Voice means challenging the *status quo*, placing stress on existing team relationships (Morrison, 2014). Trust in supervisors is a psychological state and includes positive expectations about the supervisor’s intentions or behaviors when they engage in taking risks (Gao et al., 2011). Trust in supervisors signals a strong sense of sharing within the relationship, where employees tend to exhibit new ideas and concerns without fear of being ridiculed (Chen et al., 2014). Gao et al. (2011) argued that when employees have a high level of trust in their supervisors, employees will engage in voice, even when it means taking extra risks.

Perceived rapport also involves perceptions of justice toward interaction with supervisors. When employees perceive that supervisors treat them with dignity and respect in daily encounters, they are more likely to engage in voice (Takeuchi et al., 2012). A high level of perceived rapport communicates that supervisors consider employee needs and that supervisors are willing to develop and maintain an ongoing relationship with them (Tyler and Lind, 1992). In such a context, employees are likely to be broadly and deeply invested in this relationship, and work beyond their formal job descriptions in return. For example, this may involve initiating changes in organizations (George and Brief, 1992; Zhang et al., 2015).

Thus, we hypothesized the following:

Hypothesis 3. Day-level perceived rapport relates to day-level employee voice.

Supervisor developmental feedback provides valuable information to employees, fostering their development in organizations and job-related improvements. This feedback helps create a positive team climate, free from pressure and fear of making mistakes (Zhou, 2003; Li et al., 2011). Thus, in daily encounters, supervisor developmental feedback nurtures employee perceptions of justice and trust in supervisors, enhancing perceived rapport. In turn, when employees develop perceived rapport based on a supervisor’s demonstration of developmental feedback, the relationship becomes more of a social exchange. Once they regard their relationships with their supervisors as beyond the traditional economic exchange relationship, they are more likely to reciprocate the supervisor developmental feedback. This is done by expressing constructive suggestions and concerns, oriented toward facilitating organizational effectiveness. Zhang et al. (2015) found that supervisor expression of genuine and holistic concerns toward employees develop high-quality relationships that encourage discretionary expressions of employee voice. Following the logic of SET, we developed the following hypothesis:

Hypothesis 4. Day-level perceived rapport mediates the relationship between day-level supervisor developmental feedback (morning) and day-level employee voice.

### Mediating Role of Positive Affect

#### Supervisor Developmental Feedback and Positive Affect

Positive affect is a relatively short-lived positive evaluative state, with neurological and cognitive elements (Lawler and Thye, 1999). Positive affect is immediate, internal, and involuntary. It is produced by the results of a social exchange process (Weiner, 1985). Beneficially initiating social exchange actions can engender positive affect (Cropanzano et al., 2017).

When receiving developmental feedback, employees are likely to be immersed in their current jobs, pay attention to skill mastery (Zhou, 2003), and capture potential opportunities to achieve career growth in organizations. Increasing expectations of career success in organizations enhances employees’ positive affect in the workplace (Kidd, 2004).

Moreover, supervisor developmental feedback is meant to enhance employees’ learning and development in the future (Li et al., 2011). This also signals employees that their supervisors support and care about the benefits they provide the organization. From a social exchange perspective, supervisors’ expressions of sincere concerns about followers’ personal welfare will induce employees’ positive affect in the relationship (Hsiung, 2012). Fredrickson (2003) suggested that positive affect occurs in safe and comfortable circumstances. Heled et al. (2016) discovered a positive relationship between beneficial team climate and employees’ positive emotional state. Integrating arguments above, we hypothesized the following:

Hypothesis 5. Day-level supervisor developmental feedback (morning) positively relates to day-level positive affect (morning).

#### Positive Affect and Employee Voice

Morrison (2014) suggested that voice often stems from automatic processes. Voice is consistently preceded by an intense emotional episode. Individual affect plays a vital role in shaping employee voice.

From a “broaden and build” perspective, positive affect expands individual thinking, attention, and behavioral repertories. This helps employees build social and psychological resources (Fredrickson, 2003). Positive affect provides employees with more flexible cognition and the ability to integrate diverse materials (Isen, 1998). When experiencing positive affect, employees are more likely to connect and integrate divergent stimulating content and produce more creative solutions to organization problems (George and Brief, 1992). Positive affect strengthens employees’ abilities to express their voice.

From an affect infusion perspective, positive affect drives the individual to regard the workplace as safe and to believe that everything is going well (Liu et al., 2017). Voice is risky and costly, and it can only be practiced strategically (Ng and Feldman, 2012). An employee can only engage in voice if they feel psychologically safe (Liang et al., 2012). Research has shown that employees experiencing positive affect believe favorable outcomes are more likely, and perceive others more positively (Liu et al., 2017). In such context, employees do not fear punishment when the expression of voice does not play out as desired.

Social exchange theory proposes that positive affect is an internal self-reinforcing stimulus, leading employees to reciprocate the socioemotional benefits their supervisors offer in the social exchange relationship (Lawler, 2001). This motivates employees to go beyond their job roles, engaging in extra-role behavior that enhances organization effectiveness (Chen et al., 2014). In addition, positive affect provides a greater ability to express voice. Based on this logic, we hypothesized the following:

Hypothesis 6. Day-level positive affect (morning) positively relates to day-level employee voice.

Hsiung (2012) found that when working with a supervisor who is more willing to share information and express their thoughts, employees are more likely to experience positive moods. This contributes to strengthening employee voice. Supervisor developmental feedback offers guidance to employees about how to achieve career growth in organizations, nurturing their positive affect. In turn, positive affect acts as a subtle signal to employees about their active response in this social exchange interaction. Given an increased ability and motivation to exercise voice, employees are motivated to render a constructive voice, benefitting broader organization goals. Therefore, we propose the following hypothesis:

Hypothesis 7. Day-level positive affect (morning) mediates the relationship between day-level supervisor-provided developmental feedback (morning) and day-level employee voice.

## Materials and Methods

### Samples and Procedure

Given the research purpose, this study adopted the experience sampling method (ESM) to collect data for analysis. We recruited our participants through alumni networks of our college. We first selected 187 of the alumni who volunteered, who updated their contact information and work background within the past 2 years. We then randomly chose 151 participants who worked full hours per week in China. We coded them from 1 to 151 and applied a random number generator in Microsoft Excel to choose 100 initial participants to invite for the survey process. We contacted participants through email, social apps, and telephone to request study participation. Of the initial 100 participants, 69 alumni confirmed their participation in our research. We explained the research purpose and procedure, and then formed a research group on WeChat, a social app.

On the first Sunday of the sampling period, we sent study participants a website link to our initial questionnaire (including code, gender, education, and tenure). To better infer the causal relationship between our focal variables, we collected data from two time points (e.g., 10:00 A.M. and 17:00 P.M.) according to the research of Qin et al. (2018). From the following Monday to Friday, participants received a questionnaire at 10:00 A.M. (supervisor developmental feedback and positive affect) and a questionnaire at 17:00 P.M. (perceived rapport with supervisors and employee voice). Based on the potential influences of positive affect on perceived rapport with the supervisor (Lawler, 2001), we provided the survey related to positive affect in the morning, and the survey of perceived rapport in the afternoon. Using this process, we collected 310 matched data nested across 62 people for 5 consecutive days. Five participants failed to finish the initial survey.

Study participants worked in a variety of industries in mainland China, including financial services, Internet companies, and manufacturing. This ensured sample representativeness. Male participants represented 48.4% of all subjects; 11.3% held degrees of college and below; 41.9% held bachelor degrees; and 46.8% held master’s degrees and above. The mean tenure in the current organizations was 8.218 (*SD* = ±7.602).

### Measurement

A self-reported five-point Likert scale was used in the study: 1 indicated “strongly disagree” and 5 indicated “totally agree,” if there was not a special explanation. All items were translated into Chinese using a back translation procedure (Brislin, 1970), ensuring translation accuracy.

#### Supervisor Developmental Feedback

We used the three-item scale developed by Zhou (2003) to measure supervisor developmental feedback. A sample item was “Today my supervisor focused on helping me to learn and improve while giving me feedback.” The Cronbach’s α for this scale was 0.941.

#### Voice

The four-item voice scale, developed by Van Dyne and LePine (1998) based on the research of Lebel (2016), was used to measure employee voice. A sample item was: “Today I spoke up with concerns about work not being done effectively.” The scale yielded a Cronbach’s α of 0.871. The study adopted self-reported questionnaires, rather than supervisor-reported questionnaires, to measure voice. This is because in ESM, it is difficult for supervisors to differentiate employee voice on a daily basis (Carpenter et al., 2014).

#### Positive Affect

The 20-item Positive and Negative Affect Schedule (PANAS) scale was adopted to measure daily positive affect. The current adopted five items with highest loadings in the positive affect scale (e.g., Enthusiastic, Interested, Determined, Excited, and Inspired). The Cronbach’s α for this scale was 0.909.

#### Perceived Rapport

We adapted four items from the rapport dimension of the Subjective Value Inventory (SVI), developed by Curhan et al. (2006). The four items were “Did your supervisor consider your wishes, opinions, or needs today?”; “Do you feel your supervisor listened to your concerns today?”; “How satisfied are you with your relationship with your supervisor as a result of the communication today?”; and “Does the communication today make you trust your supervisor?” Items were rated on a five-point scale, ranging from “1 = not at all” to “5 = a great deal.” The Cronbach’s α of this scale was 0.927.

#### Control Variables

We incorporated gender (coded as 0 for male and 1 for female), education (coded as 1 for college and below, 2 for bachelor, and 3 for master and above), and tenure in the current organization (years), based on their potential influence on our results (Hsiung, 2012; Duan et al., 2017; Qian et al., 2018). Following the suggestion of Aguinis and Vandenberg (2014), we conducted separate analyses with and without the control variables. The results were virtually the same, and removing the control variables from the equation models did not alter the interpretation of the findings. Thus, we reported the results without control variables in this study.

### Analytical Approach

We collected data at the between-person level (gender, education, and tenure) and at the within-person level (supervisor developmental feedback, positive affect, perceived rapport, and employee voice) from our samples. Considering the nested nature of our data, we adopted multilevel data modeling (Raudenbush and Bryk, 2002) for the analysis. We applied Mplus 7.4 (Muthén and Muthén, 1998/2012) software, using a restricted maximum likelihood estimation method for the analysis. Based on previous research, the multilevel path analysis was effective to test our conceptual model (Koopman et al., 2016), because it permitted a simultaneous examination of the underlying affect (i.e., positive affect) and relationship (i.e., perceived rapport) paths through which daily supervisor developmental feedback impacted employee voice.

The first stage of hypothesis testing was to investigate the systematic within- and between-person variance for the daily variables. The proportion of within-person variance for voice was 23%; for supervisor developmental feedback, it was 26%; for positive affect, it was 34%; and for perceived rapport, it was 29%. These results justified the use of multilevel analysis. We ran the multilevel path analysis with multiple mediators with random slope and used robust estimators indicated the within-person effect. Before the regression analysis, all the daily variables were group centered.

## Results

### Confirmatory Factor Analysis

For the self-reported questionnaire, we adopted a multilevel confirmatory factor analysis (MCFA) to test the validity of the four-factor conceptual model, before conducting hypothesis testing. Table 1 shows that the hypothesized four-factor model was a better fit with the data, compared with other models.

**TABLE 1:** Results of multilevel confirmed factor analysis.

**Model**	**Variables**	**χ^2^**	***df***	**Δχ^2^**	**RMSEA**	****RMR****	****CFI****
Four-factor	SDF, V, PA, PR	320.306	98		0.086	0.051	0.944
Three-factor	SDF + V, PA, PR	794.631	101	474.325^∗∗^	0.149	0.108	0.827
Three-factor	SDF + PA, PR, V	1027.590	101	707.284^∗∗^	0.172	0.120	0.768
Three-factor	SDF + PR, PA, V	867.789	101	547.483^∗∗^	0.156	0.085	0.808
Three-factor	SDF, V + PA, PR	815.656	101	495.350^∗∗^	0.151	0.124	0.821
Three-factor	SDF, V + PR, PA	737.140	101	416.834^∗∗^	0.143	0.103	0.841
Three-factor	SDF, V, PA + PR	1182.700	101	862.397^∗∗^	0.186	0.149	0.730
One-factor	SDF + V + PA + PR	1934.400	104	1614.09^∗∗^	0.238	0.147	0.542

*SDF, supervisor developmental feedback; V, employee voice; PA, positive affect; PR, perceived rapport. ^∗∗^*p* < 0.01.*

### Multilevel Path Analysis Results

Table 2 provides the results of descriptive statistics and intra-correlations among the focal variables in this study. Supervisor developmental feedback positively related to voice (*r* = 0.335, *p* < 0.01), positive affect (*r* = 0.415, *p* < 0.01), and perceived rapport with supervisor (*r* = 0.479, *p* < 0.01). Voice positively related to positive affect and perceived rapport with supervisors (*r* = 0.301, *p* < 0.01). Positive affect positively related to perceived rapport with supervisors (*r* = 0.254, *p* < 0.01).

**TABLE 2:** Means, standard deviations, and correlations.

**Variable**	**Mean**	***SD***	**1**	**2**	**3**	**4**	**5**	**6**	**7**
1. Gender	0.520	0.501							
2. Education	2.350	0.675	−0.113						
3. Tenure	8.218	7.602	0.247	−0.337^∗∗^					
4. Voice	3.518	0.750	−0.030	−0.100	0.056	(0.871)	0.335^∗∗^	0.278^∗∗^	0.301^∗∗^
5. Supervisor developmental feedback	3.241	0.980	−0.021	−0.049	−0.116	0.534^∗∗^	(0.941)	0.415^∗∗^	0.479^∗∗^
6. Positive affect	3.076	0.850	−0.220	−0.216	0.028	0.534^∗∗^	0.572^∗∗^	(0.909)	0.254^∗∗^
7. Perceived rapport	3.351	0.800	0.125	−0.103	−0.032	0.590^∗∗^	0.751^∗∗^	0.526^∗∗^	(0.927)

*Correlations below the diagonal are at between-person level (i.e., for each employee scores were averaged across the 5 days during the survey); correlations above the diagonal are at within-person level. ^∗∗^*p* < 0.01. Values in the parenthesis are Cronbach’s α.*

Table 3 shows the results of multilevel path analysis with a random slope. Supervisor developmental feedback positively related to positive affect (γ = 0.312, *p* < 0.01), perceived rapport (γ = 0.292, *p* < 0.01), and employee voice (γ = 0.136, *p* < 0.05). Further, both positive affect (γ = 0.128, *p* < 0.05) and perceived rapport (γ = 0.146, *p* < 0.05) positively related to employee voice.

**TABLE 3:** Dual path mediation model test.

**Paths**	**Estimator**	**SE**	**95% LLCI**	**95% ULCI**
Supervisor developmental feedback → Positive affect	0.312^∗∗^	0.061	0.192	0.432
Positive affect → Employee voice	0.128^*^	0.065	0.001	0.255
Supervisor developmental feedback → Employee voice	0.136^*^	0.068	0.030	0.269
Supervisor developmental feedback → Perceived rapport	0.292^∗∗^	0.068	0.159	0.425
Perceived rapport → Employee voice	0.146^*^	0.065	0.019	0.273

*N* = 310 observations nested within 62 individuals. LLCI, lower level confidence interval; ULCI, upper level confidence interval. ^*^p < 0.05; ^∗∗^*p* < 0.01.

To test the robustness of the mediating effects of positive affect and perceived rapport, we used R (version 3.5.3) software to run the Monte Carlo test. Table 4 shows the results and indicates that the result of the Monte Carlo test found that both the positive affect path (effect = 0.052, 95% CI = [0.011, 0.094]) and relationship path (effect = 0.039, 95% CI = [0.006, 0.072]) were significant. In contrast, the differences between these two paths were not significant (effect = 0.013, 95% CI = [-0.030, 0.056]). The outcome further supported hypothesis 3 and hypothesis 6.

**TABLE 4:** Monte Carlo bootstrapping test.

**Effect**	**Estimator**	**SE**	**95% LLCI**	**95% ULCI**
Direct effect	0.136^*^	0.068	0.001	0.270
Indirect effect				
Supervisor developmental feedback → Positive affect → Employee voice	0.052^*^	0.021	0.011	0.094
Supervisor developmental feedback → Perceived rapport → Employee voice	0.039^*^	0.017	0.006	0.072
Difference	0.013	0.022	−0.030	0.056

*N* = 310 observations nested within 62 individuals; bootstrapping = 20,000; LLCI, lower level confidence interval; ULCI, upper level confidence interval. ^*^p < 0.05.

## Discussion

This study included the collection of daily data using an ESM for five consecutive days. The study examined the positive relationship between day-level supervisor developmental feedback and employee voice. This is consistent with and further advances previous research concerning leadership and employee voice. Leadership has always been regarded as a critical antecedent to employee voice (Detert and Burris, 2007). Within the social exchange framework, the use of employee voice depends on the treatment received from supervisors. Supervisors offer qualitative information facilitating employee development, by providing developmental feedback. To reciprocate for supervisors’ positive treatment, employees provide change-oriented suggestions. Our research provided empirical evidence for this social exchange relationship on a daily basis.

Furthermore, we examined the underlying affect and relationship paths for the relationship between feedback and voice. The result showed that positive affect and perceived rapport mediated the positive influences of supervisor developmental feedback on employee voice at the episode level. Relationship and positive affect have always been regarded as motivation factors, increasing employee enthusiasm and triggering intrinsic motivations to improve the organizational *status quo* (Hsiung, 2012). Hsiung (2012) provided empirical evidence for the mediating roles of relationship and affect within the social exchange framework. Moreover, Colquitt et al. (2013) provided meta-analytic evidence for these two paths. Based on research of Hsiung (2012) and Colquitt et al. (2013), we further tested the dual-path model, adopting a within-person approach.

Lawler and Yoon (1996) found that positive social exchange induced positive effect, which in turn increased relational cohesion and commitment behaviors. However, our research did not test the sequential mediation effects of positive affect and perceived rapport in the relationship between supervisor developmental feedback and employee voice. A potential reason for this is that important moderators may have been omitted in this study. The relationship between positive affect and perceived rapport was stronger when employee regarded supervisors’ support as stable and controllable (Lawler, 2001). Therefore, it is critical to investigate the sequential relationship between positive affect and perceived rapport with a different research design involving potential moderators (e.g., supervisor authenticity).

### Theoretical Implications

Our research contributes to the voice and SET literature in two ways. First, this study revealed the positive influences of supervisor developmental feedback on employee voice at the episode level, extending our understanding of the antecedents of voice. Given the importance of voice for both organizations and individuals, previous research focused on the relationship between supervisor behavior and voice (Mowbray et al., 2015). However, those studies have not considered the influences of supervisors’ daily interaction with employees. In a daily organization context, supervisors’ managerial strategies are implemented and transferred through interaction with their employees (Schaubroeck et al., 2016). It is important to gain insights into this dynamic process to enhance managerial effectiveness.

This study adopted supervisor developmental feedback, one of the basic supervisor interaction strategies, as an influencer of employee voice. Day-level supervisor developmental feedback enhances employees’ potential opportunities to develop and grow, by providing them with general rules about how to behave in organizations (Zhou, 2003). In addition, supervisor developmental feedback can help create a positive team climate, free from pressure and fear of making mistakes. This can minimize the psychological costs of voice (Li et al., 2011). Thus, driven by the reciprocal norm (Cropanzano and Mitchell, 2005), employees are more likely to engage in voice to improve organizational effectiveness, returning the benefits offered by supervisors. Our research provides a new perspective on how voice is integrated in the daily interaction of supervisor–employee interaction, enlarging the scope of the voice literature.

Second, this study explored both the affect and relationship paths through which day-level supervisor developmental feedback influences day-level employee voice, contributing to SET. Within the SET framework, previous research provided evidence of the mediating role of relationship and affect at the between-person level (Hsiung, 2012). However, few studies have explored these two mechanisms at the within-person level. Perceived rapport with supervisors or affect fluctuates on a daily basis. We advanced Colquitt et al. (2013) dual path social exchange model, by introducing the notion of daily within-person variations in employee positive affect and perceived rapport with supervisors. This study investigated whether the relationship and affect mechanisms that explain the employee voice associated with resource exchanges at the episode level are similar to those at the between-personal level. This adds to our knowledge of the dynamic social exchange process.

This study found that supervisor developmental feedback provides employees valuable information and a positive climate, facilitating their growth in organizations (Zhou, 2003). Day-level supervisor developmental feedback enhances employees’ positive affect and perceived rapport with supervisors. Consequently, positive affect and perceived rapport advance employee motivation and strengthen the ability to express voice. This study found no significant difference between affect and relationship path. Positive affect and perceived rapport have the same influences in facilitating the transformation from supervisor developmental feedback to employee voice. Our research advances SET by examining SET at the episode level.

### Practical Implications

This study has practical implications for managerial practices. First, daily developmental feedback by supervisors can support employees in finding their voices. In daily supervisor–employee interactions, the supervisor should provide developmental feedback, rather than simply performance feedback. However, managers should also focus on the characteristics of supervisor developmental feedback. Developmental feedback is informational in nature (Zhou, 2003). Thus, when giving developmental feedback to employees, managers should provide clear information, to enhance the positive influences associated with supervisor developmental feedback (Li et al., 2011).

Second, by considering positive affect and perceived rapport as mediators, this study revealed that managers should be alert to employees’ positive affect and perceived rapport induced by supervisor developmental feedback. Once employees’ positive and perceived rapport increases, their motivation and ability are both strengthened, encouraging them to express their voice. Thus, managers should be responsible for maintaining and enhancing their employees’ positive affect and perceived rapport.

### Limitations and Future Research

Like all studies, this research had some limitations. First, common method variance (CMV) remains a concern (Fuller et al., 2016). While the study adopted a two-wave ESM, all the data were collected from a self-report questionnaire, increasing the risk of a CMV problem. Future studies should assess voice from the perspective of supervisors or colleagues, to rule out CMV and to test the robustness of these results.

Second, we cannot infer causal relationships between our focal variables. We attempted to establish a firm causal effect between supervisor developmental feedback on employee voice using multi-wave diary analysis. However, a reverse causal effect remains a possibility. A different experimental design may support the development of firm causal results.

Third, Lawler and Thye (1999) proposed that an individual’s emotional state is the starting point of a relationship. Sears and Hackett (2011) provided empirical evidence for the positive relationship between positive affect and LMX. To consider the implicit influence of positive affect on perceived rapport, we collected data at different time points in a day. However, the current study did not provide significant support for this relationship (γ = 0.133, n.s.). This outcome aligns with findings by Hsiung (2012) and Colquitt et al. (2013), which also found that the relationship and affect paths are independent. Thus, future studies should examine potential boundary conditions to assess the relationship between these two paths.

Fourth, this research adopted a two-wave ESM design to collect data at 10:00 A.M. and 17:00 P.M. to explore the relationship between supervisor development feedback and employee voice and its underlying mechanism based on previous research. However, the daily self-report experiences (e.g., positive affect and perceived rapport with supervisors) fluctuate within a workday. Our research did not capture the influence of the variations in our focal variables. Future research could combine an ecological momentary assessment and an ESM to offer a more comprehensive insight into the dynamic social exchange process.

## Data Availability

All datasets generated for this study are included in the manuscript and/or the supplementary files.

## Ethics Statement

The study procedures were approved by the Ethics Committee of Kunming University of Science and Technology and were in line with the 1964 Helsinki Declaration and its later amendments or comparable ethical standards. Informed consent was signed and obtained from all individual participants included in the study.

## Author Contributions

ZZ was responsible for writing the manuscript. BC and LZ were responsible for data collection. VR and JZ were responsible for data analysis. All authors contributed equally to the idea development and writing of the study.

## Conflict of Interest Statement

The authors declare that the research was conducted in the absence of any commercial or financial relationships that could be construed as a potential conflict of interest.
